# Optical crosstalk and other forms of light interference in pulse oximeter comparison studies

**DOI:** 10.1007/s10877-023-01060-y

**Published:** 2023-08-23

**Authors:** Panicos A. Kyriacou

**Affiliations:** Research Centre for Biomedical Engineering, City, University of London, London, UK

**Keywords:** Pulse oximetry, Crosstalk, Light / optical interference, Penumbra effect / optical shunting, Sensor malposition, Optical shielding

## Abstract

**Purpose**: Pulse oximeter accuracy is important for the quality and safety of patient care. Methodological errors occurring during pulse oximeter accuracy studies can confound results. One potential source of error during pulse oximeter comparison studies is optical interference due to sensor-to-sensor crosstalk. Optical crosstalk can occur whenever pulse oximeter sensors are tested in close proximity of one another, as occurs during pulse oximeter comparison studies. **Methods**: This publication represents the first comprehensive review of sensor-to-sensor crosstalk and other forms of optical interference during pulse oximeter comparison studies. A review of the published literature was undertaken to elucidate the mechanism of optical crosstalk, along with other forms of optical interference, and a solution (shielding) is offered. **Results**: When pulse oximeter sensors are placed close to each other, as occurs during comparison studies, the red and near-infrared light used can also enter an adjacent sensor and lead to error. Pulse oximeter manufacturers have designed systems to reject some forms of optical interference, such as ambient light. However, light emanating from adjacent sensors during comparison studies can cause artifact, and this can be exacerbated by sensor malposition. Proper sensor placement and use of optical shielding are the best solutions to prevent crosstalk. **Conclusions**: Crosstalk and other forms of optical interference can corrupt pulse oximeter readings. Proper sensor placement and use of optical shielding of sensors are crucial steps to help protect the integrity of the data. Studies to further characterize crosstalk during pulse oximeter comparison studies are needed.

## Introduction

Pulse oximeters have been used by clinicians for decades to noninvasively measure functional blood oxygen saturation (SpO_2_) and to monitor patients’ cardio-respiratory status. Medical device pulse oximeters are regulated by government agencies, e.g., Food and Drug Administration (FDA) in the United States and Medicines and Healthcare products Regulatory Agency (MHRA) in the United Kingdom. These regulatory agencies require manufacturers to perform clinical testing on pulse oximeters to confirm performance and safety prior to receiving authorization for medical use.

The governing standard for pulse oximeters, ISO 80601-2-61, establishes that healthy human subjects undergoing induced hypoxemia are required for validating pulse oximeter accuracy, using one of two methods: (1) comparison of SpO_2_ to arterial blood analyzed by a co-oximeter, or (2) comparison of SpO_2_ to an already validated pulse oximeter. Comparison studies are not only utilized by manufacturers to acquire data required for regulatory validation; they are also used to evaluate efficacy of engineering enhancements, and by independent researchers characterizing pulse oximeter models and sensors.

**Fig. 1:**
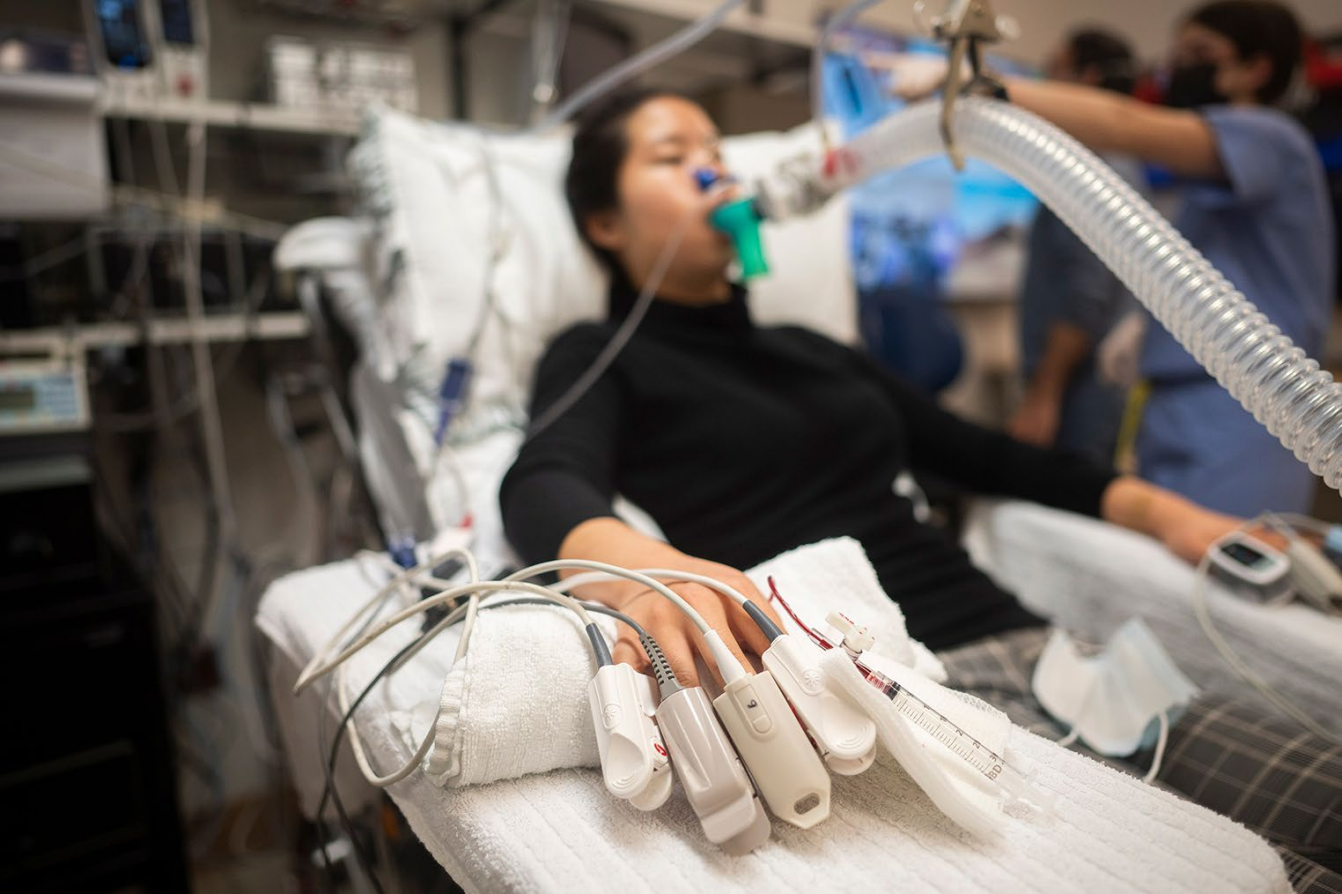
Example of multiple adjacent (unshielded) pulse oximeter sensors on both hands. This experimental configuration has a high likelihood of sensor-to-sensor crosstalk and can result in inaccurate data for both SpO_2_ and pulse rate. Image reproduced (with permission) from Usha Lee McFarling’s article, “No one’s quite sure how to fix pulse oximeters. The FDA asked this lab to find answers.” *Stat News*. Dec. 16, 2022 [1]

During the performance of comparison studies, sources of artifact must be mitigated. Crosstalk is an important source of optical interference that can occur whenever two or more pulse oximeter sensors are placed on the same extremity, or in close proximity to one another. A recent image from *Stat News* (Fig. 1) depicts a subject undergoing a pulse oximeter comparison study with multiple adjacent unshielded sensors (high risk for crosstalk). Data acquired from such a configuration should always raise the concern of experimental error due to crosstalk-mediated data corruption, which could lead to erroneous and misleading conclusions.

Despite nearly 40 years of published reports describing the effect of light interference in pulse oximetry, including crosstalk, researchers do not always avoid this important confounder when conducting comparison studies. Accounts of crosstalk began to emerge in the 1980’s when clinical observations demonstrated optical interference of pulse oximeters emanating from infrared heat lamps [2]. An early review on pulse oximeter crosstalk published in 1991 emphasized the potential hazard from infrared heat lamps, as well as from surgical lights [3].

Crosstalk has been described in the literature using alternative terms, such as ambient light interference and optical flooding. Regardless of the terminology, the mechanism involves a disruption of the photoplethysmograms (PPGs). PPGs are the pulsatile optical signals used for the estimation of blood oxygen saturation, and hence any “corruption” of the PPG waveform due to external light interference can lead to additional SpO_2_ error. In addition to erroneous SpO_2_ values, corrupted PPG waveforms can alter other parameters derived from the PPG (e.g., respiratory rate, perfusion index, and heart rate).

This document primarily reviews the principles and literature on sensor-to-sensor crosstalk during comparison studies. Crosstalk interference of pulse oximeters due to external light-emitting diode (LED) systems are also discussed due to their relevance in both clinical environments and some laboratory settings. In addition, other important forms of optical interference resulting from sensor malpositioning are discussed, including the penumbra effect and optical shunting. The important universal solution of shielding is often overlooked in laboratory comparison studies. It is also emphasized, because deployment of protective shielding can fully block the intrusion of extraneous red and infrared light from reaching a pulse oximeter detector during comparison studies.

## Methods

Searches of the literature were conducted in Web of Science, PubMed, Science Direct, Scopus, and Ovid (including journals from Ovid, CityLibrary Journals@Ovid, Allied and Complementary Medicine (AMED), Embase, Global Health, and Ovid MEDLINE). Keywords used in this search included: “Pulse oximetry”, “crosstalk”, “light interference”, “optical interference”, “optical shunting”, “penumbra”, “light flooding”, “ambient light”, “infrared heat”, “sensor malposition” and “sensor misalignment”. Database searches yielded 71 results. A manual search of all the relevant papers was carried out to identify papers investigating the effect of light in the performance of pulse oximeters. Following full-text analysis, 14 studies met the inclusion criteria and were included in this narrative review.

## Sensor-to-sensor crosstalk

In understanding sensor-to-sensor crosstalk, one must recognize that infrared light travels through some tissues more easily than red light, and it can also reflect from nearby hard surfaces (e.g., equipment, walls, and floors) without being seen. Any nearby source of infrared light can cause optical interference without being directly aimed at the pulse oximeter sensor. Therefore, even if the red light of a sensor is not visibly shining on an adjacent sensor, the risk of optical interference from crosstalk is still present. It is prudent to assume crosstalk will occur whenever two or more pulse oximeter probes are placed adjacent to one another.

The mechanism of optical “crosstalk” requires additional study, but it is hypothesized to result when stray light emanating from a source other than the sensor’s emitter is being sensed by the pulse oximeter’s detector (mainly photodiodes). In the example shown in Fig. 1 above, the contaminating source would be the red/infrared light projecting from adjacent unshielded pulse oximeter sensors. Optical crosstalk could lead to corruption of the PPG waveform and can result in an erroneous SpO_2_ value. Crosstalk errors can originate from several sources, such as the reflection of light from nearby surfaces or the presence of other light sources in the ambient environment. Crosstalk error severity depends on the intensity of the contaminating light, as well as the temporal overlap and wavelength spectrum of the contaminating light.

To fully understand the significance of crosstalk as a confounder in comparison studies, it is important to review some basics on how pulse oximeters work. Pulse oximeters measure the ratio of pulsatile (arterial signal) to non-pulsatile red and infrared light absorption at two or more specific wavelengths. Oxyhemoglobin (peak absorption 940 nm) absorbs more infrared light than deoxyhemoglobin, and deoxyhemoglobin (peak absorption 660 nm) absorbs more red light than oxyhemoglobin [4]. Red and infrared LEDs from one side of the probe shine light through the tissue to a photodetector on the opposite side of probe, which senses the red and infrared light that has been absorbed or reflected by the pulsating arterial blood. Thus, pulse oximeters depend on time-varying light signals to detect arterial blood absorbance changes in the measurement site. Any disturbance of the PPG signal due to external light in the red to near-infrared spectrum can cause erroneous SpO_2_ values.

It is recommended that pulse oximetry sensors be positioned according to the manufacturer’s directions for use (DFU) to avoid potentially interfering ambient light reaching the photodetector. In addition, manufacturers typically employ engineered electronic filtering solutions to alleviate some impact of ambient light interference. However, such filtering techniques, irrespective of how advanced they are or claim to be, cannot guarantee the absolute elimination of light interference. Hence, any light component in the red to the near-infrared range of the spectrum can still enter the sensor if not optically shielded. Most pulse oximeters operate at similar wavelengths (660 and 940 nm) and use LED multiplexing. Therefore, interference between devices should be anticipated in a scenario where sensors are placed in proximity of one another, since each pulse oximeter could receive additional light from the adjacent sensor. Again, ambient light shields can effectively prevent the effects of stray light sources, provided they also block infrared light.

Sensor-to-sensor crosstalk is far more likely to occur during comparison testing of pulse oximeter sensors, as they are frequently deployed on adjacent application sites (e.g., fingers), a situation which is not as common in the clinical environment. Since the adjacent sensor setup is almost completely relegated to research environments, peer-reviewed reports analyzing factors affecting crosstalk in this scenario have not yet been published. However, because comparison studies are used to draw clinical conclusions on the effectiveness of pulse oximeters, further prospective laboratory studies are needed to quantify the degree of error caused when various sensors are used independently or properly shielded, versus when used in close proximity to one another.

## External light interference

There are several scenarios (in the clinic as well as the lab) where external light interference can be encountered when using pulse oximeters. Such interference can emanate from ambient light, as well as from specialized medical devices that use optical systems. This section reviews external light interference from ambient light as well as from specialized optical equipment, including infrared heaters and communications systems, operating room lights, and surgical navigation systems.

### Ambient light

Pulse oximeter designs have long included the ability to reject ambient light in their processing, evident by the fact that standard pulse oximetry test equipment, such as the popular Fluke Biomedical Patient Monitor Simulators (Fluke Corporation, Washington, US), have the ability to simulate light interference sources, including sunlight, DC, 50 Hz/60 Hz and pulsed 1–10 Hz.

During the early adoption of pulse oximeter technology, clinicians reported situations where pulse oximeters became unreliable [2], and simple solutions were quickly developed [5]. Subsequently, investigators such as Fluck, et al. studied the errors introduced by various types of lighting commonly used in hospitals [6]. These included incandescent, quartz-halogen, infrared, fluorescent, and bilirubin lights (blue/violet light therapy lamps). Those investigators concluded that common light sources used in hospital settings had minimal impact on pulse oximeter readings or accuracy. This suggested that ambient light had been fully accounted for in the design of modern pulse oximetry systems and was no longer an issue. However, certain lighting technologies that use time-varying light signals can still threaten pulse oximeter accuracy, primarily LED-based hospital illumination technologies and some specialized equipment described below.

### Specialized optical medical devices

#### Infrared heaters / infrared communication

The earliest published reports of optical crosstalk in pulse oximetry were in reference to infrared heat lamps [2]. In clinical care, heating lamps are critical for temperature management during anesthetic care, especially in pediatrics [7]. However, heat lamps produce high-intensity ambient light that can prevent the pulse oximeter from sensing light transmitted through tissue to calculate the SpO_2_ value. This problem can easily be resolved by shielding the pulse oximeter sensor with a protective material that will prevent external light from reaching the detector [2, 3, 7].

#### Operating room surgical lights

While early accounts of crosstalk reported interference from surgical lights and infrared heat lamps [2, 3], a more recent case report described pulse oximeter interference caused by newly installed LED surgical lights, which resolved when moving the light away from the oximeter sensor [8]. The authors found that the interference gradually increased as the light source was moved toward the sensor, resulting in false readings, low-signal warnings, or no readings from the pulse oximeter. While modern pulse oximeters are designed to reject ambient light to minimize light interference, a modulated ambient light source can create a pulsed waveform that overpowers the PPG waveform [8].

#### Surgical navigation systems

**Fig. 2:**
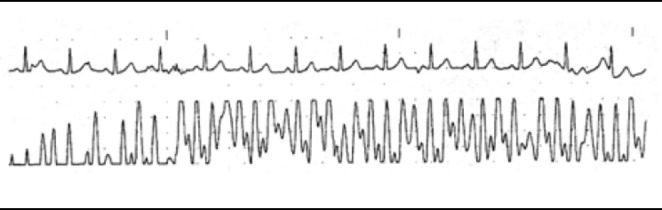
Pulse oximeter plethysmogram with interference from StealthStation surgical navigation system (bottom) and electrocardiogram (top). Reproduced (with permission) from the van Oostrom, et al. study [9]

Optical interference affecting pulse oximetry has also been reported during use of surgical navigation systems, including the StealthStation™ (Medtronic, Memphis, TN) and Brainlab neuronavigation system (Brainlab, Inc., Westchester, IL) [9–12]. The authors reported interference patterns in the pulse oximeter’s signal, false readings, or both [9–12]. One study using the StealthStation system noted a 4 Hz disturbance in the pulse oximeter’s waveform, which caused a sudden drop in displayed oxygen saturation (SpO_2_) values, as shown in Fig. 2 [9]. The StealthStation system radiates infrared light at 4 Hz, which demonstrates the effect of time-varying light on a pulse oximeter waveform.

### Sensor malposition: penumbra effect / optical shunting

Pulse oximeters rely on absorption of red and infrared LED light through the pulsatile arterial vascular bed to estimate arterial oxygen saturation. This arterial pulsation is a small percentage of the total amount of light transmitted. Thus, it is important that the light is adequately coupled into the tissue and collected thereafter by the detector. When sensors are improperly placed (i.e., not in accordance with the DFU), the reliability of the readings is compromised. This has been referred to in the literature as the penumbra effect or optical shunting. These phenomena can result from: (1) misalignment of the emitter and detector, (2) partial coverage of tissue between emitter and detector, causing a disproportionate amount of light added to the detected signal without passing through pulsatile tissue, or (3) inadequate coupling (gaps) between the emitter, tissue bed, and detector, which causes optical shunting and therefore reduces the signal-to-noise ratio. All the above have been shown to contaminate the red/infrared ratio in an unpredictable manner, leading to either higher or lower than normal SpO_2_ values [13, 14]. While perfusion index (PI) could be a quality index that could relate to both sensor malposition and critical hypoperfusion, it will be almost impossible to solely use the PI as an indicator of probe malposition.

A report by Kelleher, et al. in 1989 is the first known publication indicating that penumbra and optical shunting can be sources of error in SpO_2_ measurements [13]. In this article, erroneous SpO_2_ readings from a surgical case are described where simple repositioning of the SpO_2_ probe resulted in disappearance of the error. This observation motivated a formal investigation of malpositioned SpO_2_ probes in 20 subjects [13]. The researchers altered the penumbra by repositioning a pulse oximeter probe in intervals of 1 mm (from + 11 mm to -9 mm) from the proximal end of the fingertip on the index finger of the left hand. The protocol was repeated at ambient temperatures of 13 and 21 °C to accommodate vasoconstriction and vasodilation. The results suggest that pulse oximetry inaccuracies were caused by a combination of optical shunting, weak pulse signals and blood flow through cutaneous arteriovenous anastomoses. It was noted that the only source of ambient light was via fluorescent sources, and no surgical lamps were used.

**Fig. 3:**
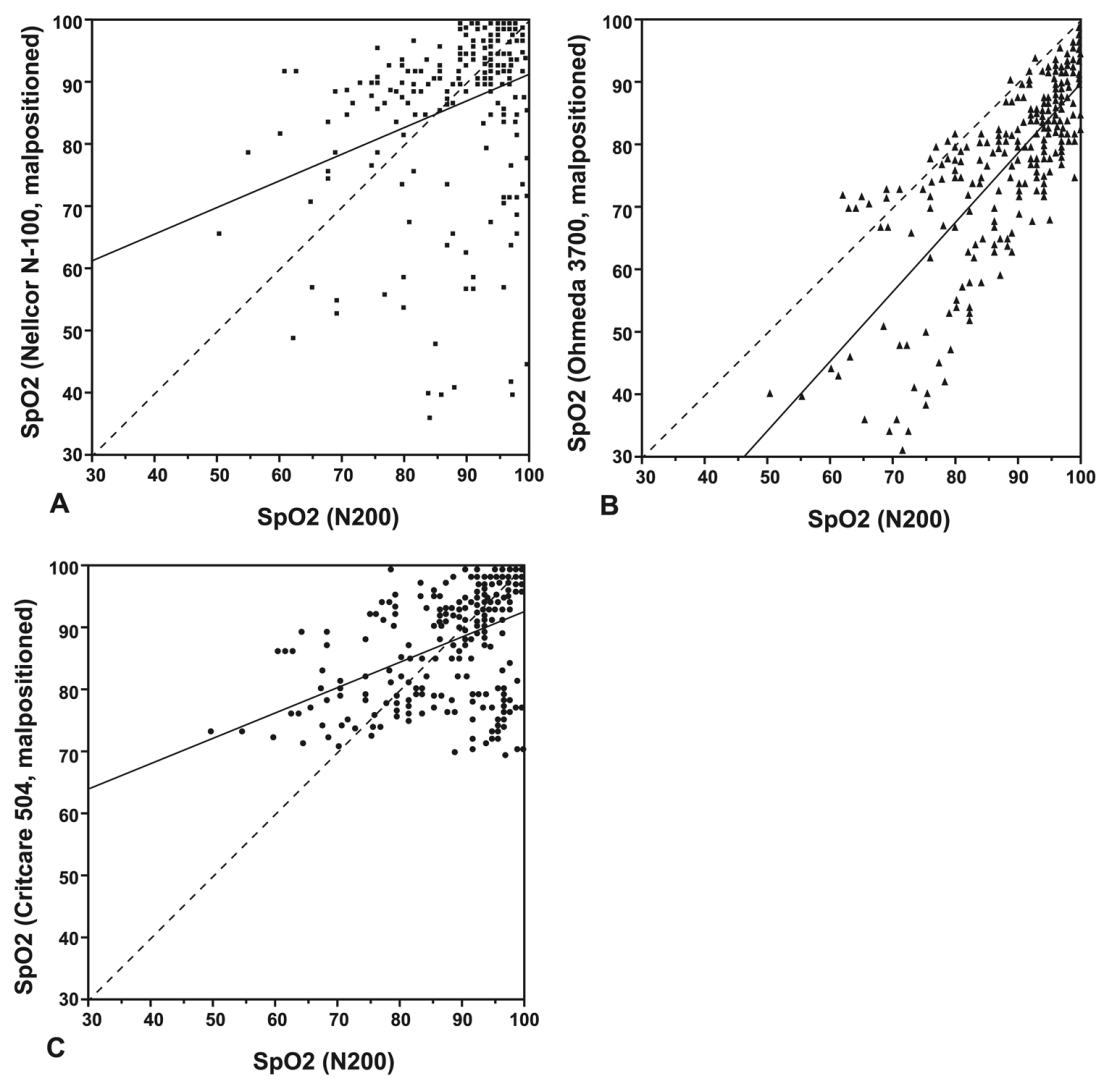
SpO_2_ values from three pulse oximeters with malpositioned sensors versus simultaneous SpO_2_ values of properly positioned control oximeter (Nellcor N-200, finger). Pooled data for 12 subjects are shown for. (**A**) Nellcor N-100, finger; (**B**) Ohmeda 3700, earlobe; and (**C**) Criticare 504, earlobe. Linear regression best-fit (solid-line) and line of identity (dashed line) are shown. Reproduced (with permission) from the Barker et al. study [14]

Investigations of this topic were confirmed in 1993 by Barker, et al., who investigated the behavior of pulse oximeters with malpositioned sensors in healthy volunteers during a controlled desaturation protocol (stepwise decrease in arterial oxygen saturation [SaO_2_] from 100 to 70%) [14]. Eight different devices were tested, with five of the eight pulse oximeter sensors malpositioned and the remaining three (properly positioned sensors) used as controls. Analysis of SpO_2_ values at five stable SaO_2_ plateaus demonstrated that the three control pulse oximeters were in agreement with the SaO_2_ samples within manufacturer specifications. However, performance of the malpositioned sensors varied greatly among different device models and SaO_2_ levels. Figure 3 shows plots of the pooled data from three of the pulse oximeters with malpositioned sensors compared to one of the control devices (Nellcor N-200). One pulse oximeter (Fig. 3B) underestimates oxygen saturation, while the other two devices (Fig. 3A and C) underestimate oxygen saturation at high SaO_2_ values and overestimate it at low SaO_2_ values.

**Fig. 4:**
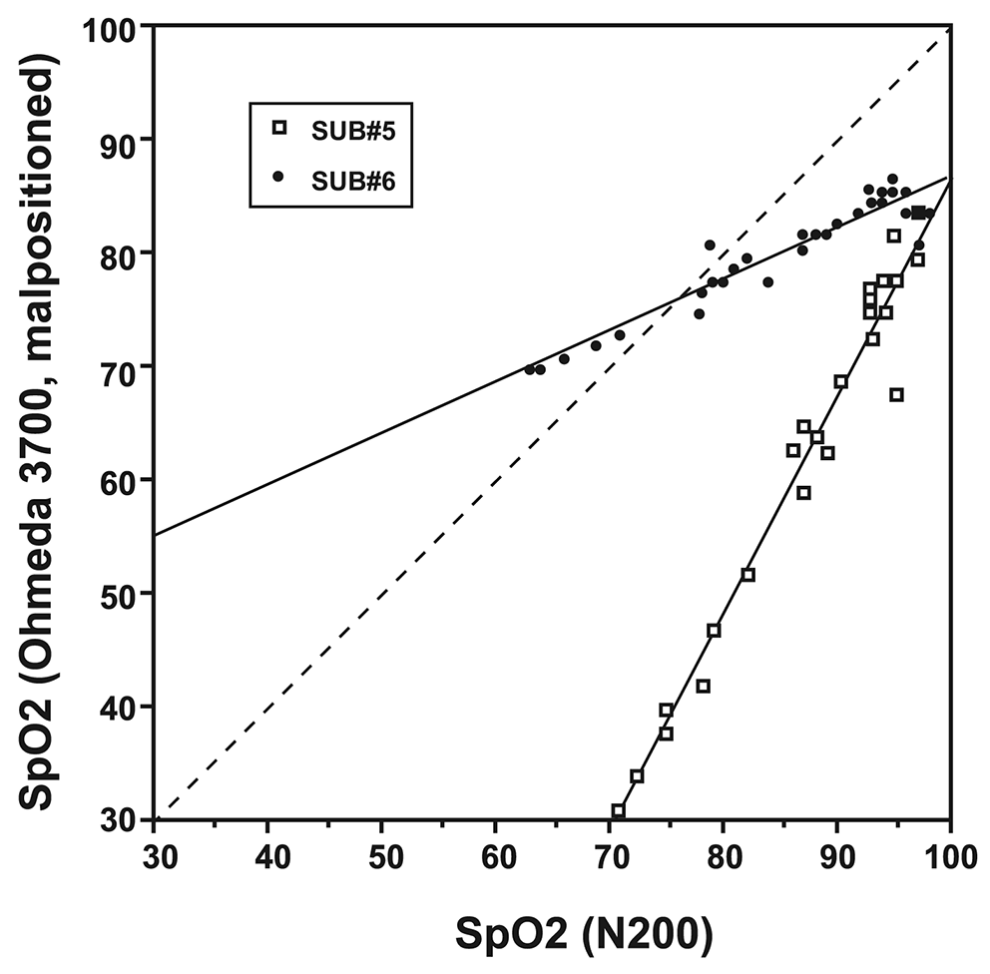
SpO_2_ values for malpositioned Ohmeda 3700, earlobe versus control SpO_2_ values; single-subject data showing two individuals and their separate linear regressions. Single-subject data fall much closer to regression lines than pooled data (Fig. 4), but the regressions for different individuals vary widely. Reproduced (with permission) from Barker et al. study [14]

In addition, there was also substantial variation in the performance of malpositioned sensors between subjects, as shown in Fig. 4 below.

The wide variation of pulse oximeter accuracy during sensor malposition underscores the importance of proper sensor placement to prevent penumbra effect errors.

### Solutions for optical interference

As mentioned earlier, manufacturers have engineered signal-filtering solutions to eliminate common sources of ambient light interference (e.g., blocking 50 and 60 Hz artifact present in European and American electrical grids, respectively). One company, Masimo (Irvine, CA), has also provided signal-filtering solutions to eliminate artifacts emanating from the harmonics of 50 and 60 Hz signal. However, solutions to mitigate ambient light interference do not prevent sensor-to-sensor crosstalk, which can still impair pulse oximeter signals when two sensors are located in close proximity without shielding.

**Fig. 5:**
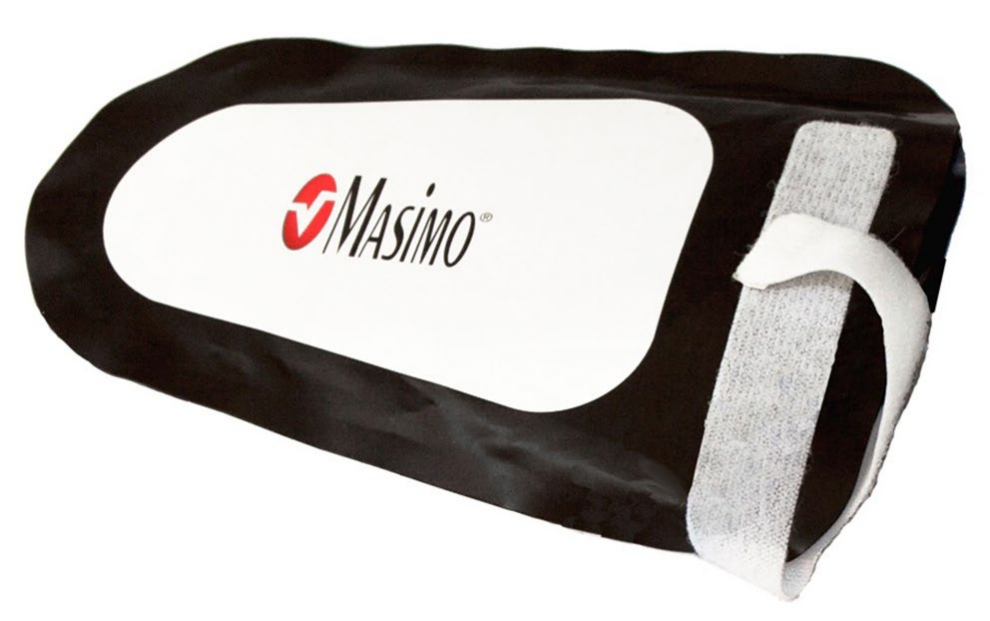
Masimo Ambient Shield Example of a Red and Infrared Light Shield. The Masimo Ambient Shield accessory is a disposable shield designed to filter out ambient light interference

When multiple sensors are used simultaneously on the same subject, the above referenced filtering solutions are not sufficient due to optical crosstalk between the sensors. In these examples (mostly occurring in experimental settings) optical shielding is required. Some investigators have used materials such as aluminum foil 3,9–12]. However, one manufacturer, Masimo, has created a commercially available accessory called “Ambient Shield” shown in Fig. 5.

Use of opaque shielding around sensors to prevent interference from modulated surgical lights and infrared heat lamps has been described in the literature for decades [3, 7]. Furthermore, the increasing reports of pulse oximeter interference from surgical navigation systems have also shown that crosstalk was resolved when proper shielding was placed around the sensor [9–12]. In one study evaluating the effect of the Brainlab neuronavigation system on six different pulse oximeters, the authors assessed two different shielding techniques, which included a cotton blanket or aluminum sheets wrapped around the pulse oximeter probe [10]. Results demonstrated a reduction in the interference pattern using either method, but the aluminum wrap achieved undisturbed saturation recognition in all subjects with almost all monitors. Using an opaque shield designed for pulse oximeter probes, such as the Masimo Ambient Shield accessory (Fig. 5), will also eliminate this interference, as demonstrated by the 2017 case report on interference from the StealthStation surgical navigation system [12].

### Clinical implications of optical interference

Although the scenarios producing optical interference in pulse oximetry discussed in sections II through IV (above) primarily emphasized problems in comparison studies, these lessons also have relevance during patient care. Medical management decisions in the clinical domain often utilize data from pulse oximeters. Despite widespread use of pulse oximeters, the problems of crosstalk and optical shunting are not widely known in the clinical community. It is important for clinicians and researchers alike to be aware of these issues, carefully follow the manufacturer’s DFU, ensure proper sensor size and placement, and utilize proper shielding in situations where crosstalk or light interference is possible.

**Fig. 6:**
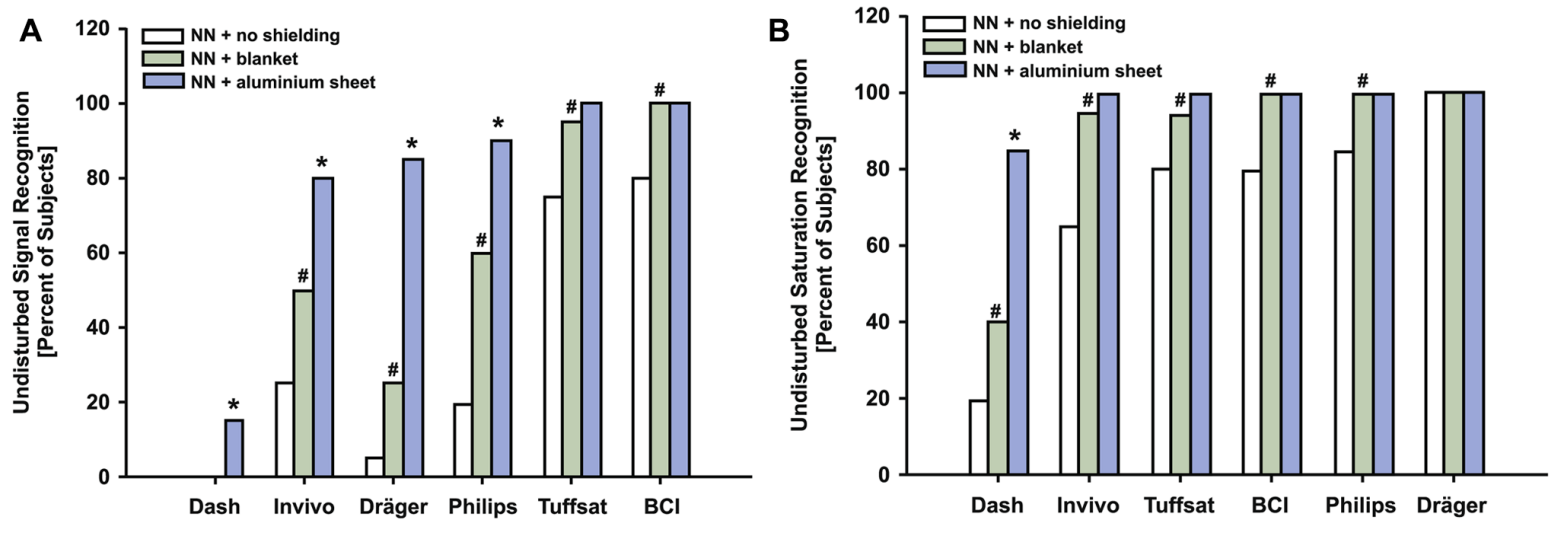
NN = neuronavigation; a pound sign (#) indicates p < 0.01 versus NN without coverage; an asterisk (*) indicates P < 0.01 versus blanket coverage. (**A**) Percentages of subjects with undisturbed signal recognition. Performance of pulse oximeters was different among manufacturers, irrespective of the method of shielding during NN (p < 0.001). (**B**) Percentages of subjects with undisturbed saturation recognition. Performance of pulse oximeters was different among manufacturers (p < 0.001). Reproduced (with permission) from the Mathes et al. study [10]

A specific example of crosstalk from an external infrared source (Brainlab neuronavigation system) was investigated for interference on the performance of six pulse oximeters from five different manufacturers. The results demonstrated significant differences among these pulse oximeters in signal quality and oxygen saturation detection (p < 0.001) with and without two different shielding methods, as shown in Fig. 6 (A and B) [10]. This shows that the crosstalk caused by interfering light sources can create large errors across various manufacturers/designs.

While the specific example of interference from the BrainLab neuronavigation system was studied, several other scenarios can influence the error introduced by optical crosstalk, depending on sensor geometries, interference band overlap, proximity to interference source, shunting pathway, saturation state of patient, and other factors. Thus, these potential errors can be highly variable and dependent on the measurement scenarios, leading to inaccurate outcome of study objectives.

### Eye to the future

Several methods have been proposed to reduce the impact of optical interference on pulse oximetry measurements, which are summarized in Table 1. The first principle is to ensure proper sensor placement per the manufacturer’s DFU. The next important concept is to use optical shielding of the pulse oximeter sensor. Shielding becomes the most important factor when multiple pulse oximeters are being evaluated in comparison studies.

**Table 1:** Summary of best practices to mitigate optical interference in pulse oximetry

	Clinical Care	Research Settings
**Sensor Position**	Carefully follow the pulse oximeter manufacturer’s DFU and ensure proper sensor size and placement	
**Sensor Shielding**	Utilize optical shielding around the pulse oximeter sensor when specialized optical medical devices are in use (e.g., infrared heaters, LED surgical lights and navigation systems)	Place optical shielding around each sensor when two or more pulse oximeter sensors are placed adjacent to one another (i.e., during pulse oximeter comparison studies)

Another supportive approach is to use a multi-wavelength pulse oximeter, which measures the absorption of light at more than two wavelengths and uses this information to separate the contributions of arterial and venous blood to the light absorption [15]. However, this system, commercially developed by Masimo as Rainbow SET®, can still be susceptible to crosstalk artifacts when numerous sensors are tested simultaneously without adequate shielding. Another approach is to use adaptive signal processing techniques, such as independent component analysis or blind source separation, to remove contaminating signals from the detected signal [16]. However, all of these signal-processing solutions are less robust than deployment of the simple optical shielding method.

Engineering and clinical studies should be pursued to seek additional solutions to reduce the effects of optical interference when multi-pulse oximeter sensor placement is utilized. By better understanding the effects of these confounders on the PPG signals, we can make further progress in eliminating SpO_2_ errors, particularly during episodes of motion and low perfusion.

## Summary

This paper provides the first substantial treatment of sensor-to-sensor crosstalk during comparison studies. Earlier publications on crosstalk almost exclusively dwell on optical interference resulting from time-varying light signals emanating from infrared heat lamps, surgical lights or navigation systems.

Errors in pulse oximeter accuracy due to ambient light sources have been largely overcome by manufacturers. However, errors can still occur from newer LED-based lighting technology during clinical care and in clinical studies involving more than one pulse oximeter sensor.

Studies have shown that the degree of optical interference from external sources and sensor malposition can differ among pulse oximeter brands depending on their placement, design, signal-processing, and how they manage the uncertainty in SpO_2_ readings [10, 14]. This has substantial implications in comparison studies where pulse oximeter sensors are placed adjacent to one another.

While these studies are typically limited to the research environment, it is crucial that the protocol includes proper sensor placement and use of appropriate shielding around each sensor to prevent optical interference. Numerous studies have shown that optical sensor shielding can resolve light interference [3, 7, 9–12]. This simple step in the study methodology will help protect the integrity of the results and ensure that accurate data are reported to the clinical community. Studies to fully characterize the problem of crosstalk during pulse oximeter comparison studies are needed.

## References

[1] McFarling UL. No one’s quite sure how to fix pulse oximeters. The FDA asked this lab to find answers. *Stat News*. https://www.statnews.com/2022/12/16/pulse-oximeters-hypoxia-lab-ucsf/. Accessed April 14, 2023.

[2] Brooks TD, Paulus DA, Winkle WE. Infrared heat lamps interfere with pulse oximeters. Anesthesiology. 1984 Nov;61(5):630. 10.1097/00000542-198411000-00042. 10.1097/00000542-198411000-00042 6497015

[3] Ralston AC, Webb RK, Runciman WB. Potential errors in pulse oximetry. III: Effects of interferences, dyes, dyshaemoglobins and other pigments. Anaesthesia. 1991 Apr;46(4):291–5. 10.1111/j.1365-2044.1991. 10.1111/j.1365-2044.1991.tb11501.x 2024749

[4] Chan ED, Chan MM, Chan MM. Pulse oximetry: understanding its basic principles facilitates appreciation of its limitations. Respir Med. 2013 Jun;107(6):789–99. 10.1016/j.rmed.2013.02.004. Epub 2013 Mar 13. 10.1016/j.rmed.2013.02.004 23490227

[5] Siegel MN, Gravenstein N. Preventing ambient light from affecting pulse oximetry. Anesthesiology. 1987 Aug;67(2):280. 10.1097/00000542-198708000-00030. 10.1097/00000542-198708000-00030 3605758

[6] Fluck RR Jr, Schroeder C, Frani G, Kropf B, Engbretson B. Does ambient light affect the accuracy of pulse oximetry? Respir Care. 2003 Jul;48(7):677–80. 12841858

[7] Zablocki AD, Rasch DK. A simple method to prevent interference with pulse oximetry by infrared heating lamps. Anesth Analg. 1987 Sep;66(9):915. 3619104

[8] Schulz EB, Ham JA. Light-emitting diode surgical light interference with pulse oximetry. Br J Anaesth. 2019 Oct;123(4):e490–1. 10.1016/j.bja.2019.07.002. Epub 2019 Jul 26. 10.1016/j.bja.2019.07.002 31353021

[9] van Oostrom JH, Mahla ME, Gravenstein D. The Stealth Station Image Guidance System may interfere with pulse oximetry. Can J Anaesth. 2005 Apr;52(4):379–82. 10.1007/BF03016280. 10.1007/BF03016280 15814752

[10] Mathes AM, Kreuer S, Schneider SO, Ziegeler S, Grundmann U. The performance of six pulse oximeters in the environment of neuronavigation. Anesth Analg. 2008 Aug;107(2):541–4. 10.1213/ane.0b013e31817e6778. 10.1213/ane.0b013e31817e6778 18633032

[11] Schulte TE, Ohnoutka JR, Agrawal A. BrainLAB interference with pulse oximetry during stereotactic brain biopsy. J Clin Anesth. 2012 Dec;24(8):675. 10.1016/j.jclinane.2012.03.010. Epub 2012 Nov 17. 10.1016/j.jclinane.2012.03.010 23164645

[12] Saito J, Kitayama M, Kato R, Hirota K (2017). Interference with pulse oximetry by the Stealth Station Image Guidance System. JA Clin Rep.

[13] Kelleher JF, Ruff RH. The penumbra effect: vasomotion-dependent pulse oximeter artifact due to probe malposition. Anesthesiology. 1989 Nov;71(5):787–91. PMID: 2817477. 2817477

[14] Barker SJ, Hyatt J, Shah NK, Kao YJ. The effect of sensor malpositioning on pulse oximeter accuracy during hypoxemia. Anesthesiology. 1993 Aug;79(2):248–54. 10.1097/00000542-199308000-00009. 10.1097/00000542-199308000-00009 8342837

[15] Aoyagi T, Fuse M, Kobayashi N, Machida K, Miyasaka K. Multiwavelength pulse oximetry: theory for the future. Anesth Analg. 2007 Dec;105(6 Suppl):53–S58. 10.1213/01.ane.0000268716.07255.2b. 10.1213/01.ane.0000268716.07255.2b 18048900

[16] Stetson PF (2004). Independent component analysis of pulse oximetry signals. Conf Proc IEEE Eng Med Biol Soc.

